# Percutaneous cementoplasty of lytic metastasis in left acetabulum

**DOI:** 10.3747/co.2007.95

**Published:** 2007-02

**Authors:** K. Harris, R. Pugash, E. David, A. Yee, E. Sinclair, J. Myers, E. Chow, on behalf of the Bone Metastases Site Group

**Affiliations:** * Radiation Oncology, Toronto–Sunnybrook Regional Cancer Centre, Toronto, Ontario; † Medical Imaging, Sunnybrook Health Sciences Centre, Toronto, Ontario.; ‡ Orthopaedic Surgery, Sunnybrook Health Sciences Centre, Toronto, Ontario.; § Radiation Therapy, Toronto–Sunnybrook Regional Cancer Centre, Toronto, Ontario.; || Palliative Care Initiative, Sunnybrook Health Sciences Centre, Toronto, Ontario

**Keywords:** Percutaneous cementoplasty, bone metastases, cancer

## Abstract

Minimally invasive procedures such as percutaneous cementoplasty can provide immediate pain relief and can restore mechanical stability for patients with bone metastases who are not candidates for surgery or who show resistance to radiotherapy or analgesic treatment. Here, we examine a case of percutaneous cementoplasty to treat a lytic lesion of the acetabulum from breast cancer. Good filling was observed, and no complications occurred. A research assistant recorded the patient’s scores on the Karnofsky Performance Scale, Townsend Functional Assessment Scale, and Brief Pain Inventory before surgery and at days 1, 2, and 4 and weeks 1, 2, and 4 post-procedure. Improvement in pain and walking ability was demonstrated within the first 48 hours of treatment, and that improvement remained constant throughout follow-up. These findings echo the literature, in that percutaneous cementoplasty provides immediate and long-term pain relief with few complications. We recommend that percutaneous cementoplasty be used as an additional tool for palliative treatment of patients with bone metastases.

## 1. INTRODUCTION

Bone metastases are a frequent complication in oncologic patients, often affecting the acetabulum and producing significant pain and disability^1^. Surgery is the treatment of choice for acetabular lesions^2^, but the surgery can be technically difficult when the metastasis is extensive^1^. Palliative radiotherapy has been shown to be effective in relieving pain from bone metastases^3^; however, some patients do not respond^4^, and the delay from treatment to bone strengthening may prove too lengthy for patients with extensive lytic lesions^2^. Analgesics can adequately control pain in 80% of advanced cancer patients, but systemic therapies are not without side effects^5^. In situations with architectural distortion and in patients deemed not suitable for open surgery, minimally invasive procedures such as percutaneous vertebroplasty and cementoplasty can provide immediate pain relief and can restore mechanical stability. Here, we report a case of a lytic metastasis in the left acetabulum from breast cancer; the metastasis was treated with percutaneous cementoplasty after administration of palliative radiotherapy.

## 2. CASE STUDY

An 80-year-old woman was treated with a modified radical mastectomy for stage I breast cancer in 1979. She developed chest wall recurrences in 1994 and was treated with surgical resection and postoperative locoregional radiotherapy at that time. In 2002, the patient was treated for benign anterior vertebral body compression at T12 with percutaneous vertebroplasty.

**FIGURE 1:**
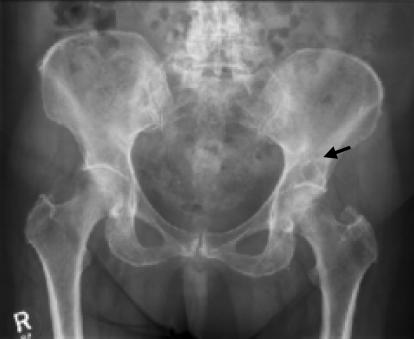
Pre-procedure plain X-ray of pelvis shows a lytic lesion within the left supra-acetabular ilium abutting the cortex.

**FIGURE 2:**
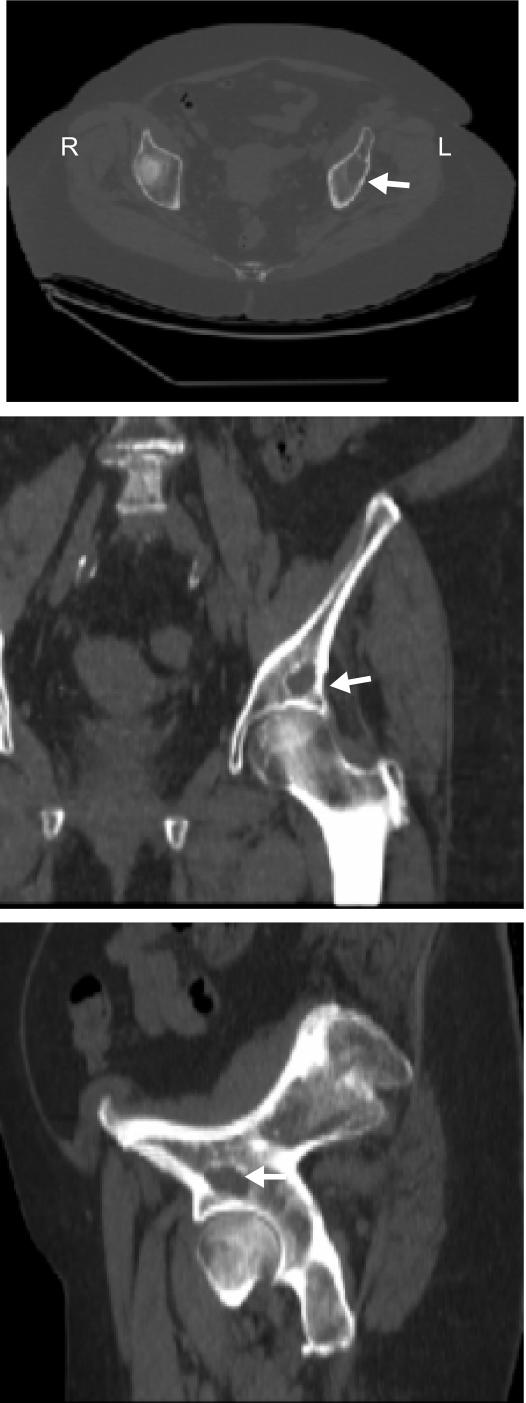
Pre-procedure computed tomography images of the pelvis show a lytic lesion in the left supra-acetabular ilium, abutting the acetabular cortex.

In April 2006, she complained of pain in her back and left hip. Plain X-ray of the pelvis revealed a lytic lesion within the left supra-acetabular ilium abutting the cortex (Figure 1). A poorly defined lesion with a non-displaced pathologic fracture was seen within the left greater trochanter. Degenerative changes within the lumbar spine and the symphysis pubis were also observed. Computed tomography (ct) images of the lumbar spine and pelvis (Figure 2) confirmed the observations and showed that the lesion in the posterior aspect of the left greater trochanter was in fact lytic and had breached through the cortex.

**FIGURE 3:**
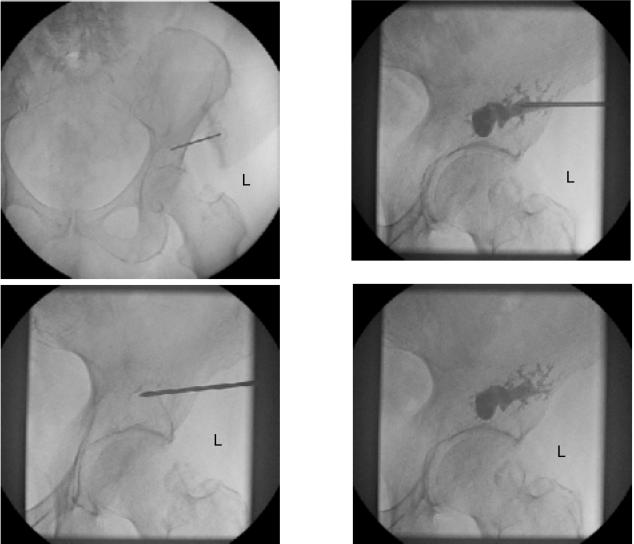
Computed tomography–guided percutaneous cementoplasty of left acetabular lytic lesion. Good filling of the left acetabular lesion was obtained, with no extravasation.

For the pain in the left hip, the patient was given a single 8-Gy fraction of palliative radiotherapy treatment that, at 1 month, had produced no significant benefit. Subsequently, the interventional radiologist and the orthopedic surgeon both recommended percutaneous cementoplasty of the left acetabulum. A 13-gauge needle [Cook M1M Osteo-Site: Cook (Canada), Stouffville, ON] was advanced into the left acetabular lesion through the anterior superior iliac spine (Figure 3). Cement (about 4.5 mL) was injected, with good filling of the left acetabular lesion and no extravasation. No periprocedural complications occurred. The lesion in the left greater femoral trochanter was left untreated.

The patient was evaluated before and after the procedure for pain, functional status, and analgesic intake. The patient rated pain intensity and functional interference according to the Brief Pain Inventory (bpi)^6^. The bpi is a patient-based assessment tool that evaluates worst, average, and current pain intensity on a scale of 0–10, with “no pain” and “worst possible pain” as descriptive anchors. Pain interference with functionality is measured in seven categories—general activity, mood, walking ability, normal work, relations with others, sleep, and enjoyment of life—on a scale of 0–10 where 0 is no interference and 10 is complete interference. Functional status was measured using the Karnofsky Performance Scale (kps) and the Townsend Functional Assessment Scale (tfas). The kps is widely used to assess functional status, level of ambulation, and ability to perform self-care in cancer patients^7^–^9^. This 11-point scale ranges from 0 to 100 in increments of 10 (where 0 means “dead” and 100 means “normal”). The tfas classifies patients into categories according to their functional capabilities^1^: “normal; pain-free use of extremity”^2^, “normal use with pain”^3^, “significant limited use” (for example, crutches, walker, cane)^4^, “non-functional extremity” (for example, use of a wheelchair or bedridden)^10^.

**FIGURE 4:**
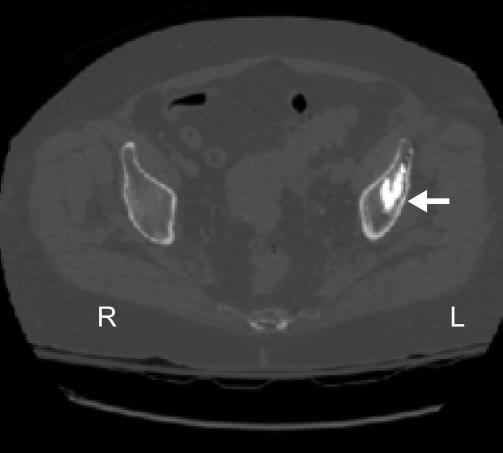
Axial computed tomography images of the pelvis and both hips show filling of the lytic lesion by cement.

Post-procedure, ct imaging of the pelvis and both hips was performed (Figure 4). Note was made of the cement that filled the lytic lesion, which extended into the base of the left ilium. Lytic lesions in the left greater trochanter and posterior iliac margin of the right sacroiliac joint were still observed.

The research assistant conducted post-procedure telephone follow-up at days 1, 2, and 4, and weeks 1, 2, and 4. Repeat tfas and bpi scores and analgesic consumption were recorded.

## 3. RESULTS

Pre-procedure, the patient had a kps score of 80, a tfas rating of 3, and a worst pain score of 4 in the left acetabulum. In the 24 hours before the percutaneous cementoplasty, the patient’s pain interfered with both walking ability and normal work at a score of 2 on a scale of 0–10, but it did not influence the other functional interference measures (score of 0 on a scale of 0–10). The patient was taking ibuprofen 6 times daily as needed for her osteoarthritic back pain.

Immediately post-procedure and at day 1, the patient’s kps, worst pain score, and functional interference remained unchanged; however, she claimed to feel dramatically better. Her tfas rating improved to a 2, because she was not relying on a cane to ambulate. At day 2, her tfas rating improved to a 1, and she reported a worst pain score of 2, with a score of 0 for the seven functional interference measures. These improved scores remained constant through the four subsequent follow-ups. No change in analgesic consumption was reported.

## 4. DISCUSSION

Deformation of the bone because of cancer may result in stress on the periosteum, causing pain^11^. When metastatic disease occurs in the pelvis, walking ability may become impaired, with consequential influence on the patient’s quality of life. Stabilization of the pelvic bone with cement injection often leads to significant pain relief and improved mobility in the treated area within 24 hours.

Weill *et al.* reported 18 patients with acetabular metastases treated with percutaneous cementoplasty^1^. In the first 72 hours, improvement was noted in 83% of cases, with 61% of patients presenting total improvement, which was defined as the disappearance of pain even while walking with no difference in analgesic medication^1^. Cotton *et al.* recorded improvement in pain in 83% of their 12 acetabular osteolytic lesion cases^12^. Both groups reported that patients with “good filling” experienced total improvement more often; however, no exact correlation between the degree of filling of the lesion and response was found^1^,^12^. Other prospective studies involving percutaneous cementoplasty treatment for bone lysis (pelvis, ilium, and femur) observed almost immediate and dramatic pain relief and improvements in ambulation for most patients^2^,^11^,^13^.

These immediate improvements in pain and walking from percutaneous cementoplasty are also long lasting. Weill *et al.* followed patients for an average of 9.4 months (range: 2–48 months) with only two incidences of pain recurrence (one at 6 months and one at 39 months), both in keeping with local tumour progression^1^. Kelekis *et al.*^13^ observed that significant pain relief persisted throughout follow-up (average: 9 months; range: 2 days to 2 years) for all of the patients who experienced immediate effects. Marcy *et al.*^2^ found that improvements in pain and walking typically extended through follow-up (average: 4.6 months; range: 11 days to 24 months).

Our findings are consistent with the cases mentioned above. The patient experienced pain relief very quickly (within 48 hours) and remained steady for the remainder of the follow-up. Functional status and interference also improved, probably as a direct result of reduced pain and improved walking ability.

Although pain relief and stabilization of the bone are the most important objectives of percutaneous cementoplasty, it is also important that complications do not arise, because they could potentially be hazardous or result in additional pain. Weill *et al.*^1^ observed cement leaks toward the soft tissue or hip joint in 28% (*n* = 5) and 22% (*n* = 4) of patients respectively. However, only 2 patients (11%) were symptomatic: one from each case of complications. Kelekis *et al.*^13^ observed leakage into the hip joint in one patient (7%), but that patient remained asymptomatic throughout the 3-month follow-up. Another patient (7%) had leakage into the obturator foramen, which led to continued pain that was managed with radiofrequency treatment of the pudendal nerve^13^. Marcy *et al.*^2^ and Hierholzer *et al.*^11^ reported no major complications and no leakage into the joint space. However, Marcy *et al.*^2^ noted that 1 patient (6%) experienced pain recurrence after the procedure because of an acetabular fracture. Although problems can arise, percutaneous cementoplasty appears for the most part to be well tolerated, with few complications.

## 5. CONCLUSION

Osseous metastases that are resistant to traditional conservative treatment modalities such as radiation or medication (or both) present a significant problem in the management of oncology patients. Surgical procedures can be dangerous for certain subsets of patients, and the undesirable effects may outweigh the clinical benefits for those with a short life expectancy^2^. Improvement in pain and walking ability is essential for enhancing patient quality of life, especially for those with good performance status and anticipated lengthier survival.

Our case echoes the literature, in that percutaneous cementoplasty of painful metastasis in the acetabulum provides immediate and long-term pain relief with few complications. We recommend that this procedure be used as an additional tool for palliative treatment of patients with bone metastases.
